# Outcomes of Femoral Neck System Procedures in a Major Trauma Centre

**DOI:** 10.7759/cureus.101113

**Published:** 2026-01-08

**Authors:** Thomas Hall, Megan Baker, Rory Padkin

**Affiliations:** 1 Trauma and Orthopaedics, Sheffield Teaching Hospitals NHS Foundation Trust, Sheffield, GBR; 2 Medical School, University of Sheffield, Sheffield, GBR

**Keywords:** avascular necrosis (avn), femoral neck fracture, femoral neck system, garden alignment index, pauwel’s angle

## Abstract

Background

Femoral neck fractures are common in elderly patients due to osteoporosis and falls. A significant complication is avascular necrosis (AVN), leading to bone tissue death and joint dysfunction. The Femoral Neck System (FNS) was developed to provide minimally invasive fixation with improved rotational and angular stability, aiming to reduce AVN risk; however, clinically significant complications still occur. Hence, this study aimed to assess complication and reoperation rates following femoral neck fracture fixation using FNS in a major trauma centre, and to compare these findings with published outcomes and alternative fixation strategies.

Methodology

A retrospective review of patients treated with FNS at Sheffield Teaching Hospitals Major Trauma Centre between 2019 and 2024 was conducted. Collected variables included demographics, comorbidities, injury mechanism, time to surgery, fracture displacement, Garden and Pauwels classification, reduction quality, and postoperative outcomes. Reduction quality was evaluated independently by two blinded orthopaedic surgeons using the Garden Alignment Index. Failures were categorised into AVN, mechanical failure, non-union, infection, or re-operation.

Results

Of the 70 eligible patients, 67 had complete data. The mean age was 73.6 years. The overall complication rate was 22.4%, with 19.4% requiring re-operation. Excluding early postoperative death, these increased to 25.9% and 22.4%, respectively. Reduction was acceptable in 79.1% of cases. Poor reduction was associated with higher complication rates (38.5% vs. 19.0%, p = 0.04). AVN represented 60% of complications, followed by mechanical failure (20%), non-union (13%), and deep infection (7%). Complications were more frequent in patients aged <65 years (43.8%) versus those aged ≥65 years (19.0%). No significant associations were identified with fracture displacement, injury mechanism, gender, or surgical timing.

Conclusions

Despite its biomechanical advantages, FNS may be associated with higher complication rates, particularly in younger patients and vertically orientated fracture patterns. Vertically aligned fractures (Pauwels III, Garden III-IV) and suboptimal reduction are strong predictors of failure. Further prospective comparative studies are required to better define the role of FNS in femoral neck fracture management.

## Introduction

Femoral neck fractures are particularly common in elderly patients due to osteoporosis and falls. A significant complication associated with femoral neck fractures, particularly with intracapsular fractures, is avascular necrosis (AVN). This typically occurs when there is a disruption to the blood supply of the femoral head. This arrest of blood flow leads to the death of bone tissue and may cause the collapse of the femoral head. As the femoral head deteriorates, it can lead to joint dysfunction, severe pain, and the eventual need for hip replacement surgery [1,2].

Complications related to AVN following femoral neck fractures can occur in up to 30% of cases [3], depending on the severity of the fracture, surgical timing, and intervention choice. Displacement or inadequate fixation increases the risk of AVN. Younger patients and those with more complex (vertically oriented) fractures are particularly vulnerable [4].

The Femoral Neck System (FNS) aims to minimise these risks by providing a minimally invasive fixation device that stabilises rotation and angulation, maintaining femoral head blood flow. Nevertheless, even with optimal surgery, AVN may still develop.

Within our major trauma centre, a large number of patients undergoing FNS appear to develop AVN. This prompted a review of all FNS cases performed from 2019 to 2024, with detailed evaluation of fracture classification according to Pauwels and Garden classification systems [5,6], reduction quality, complication causes, and comparison to published outcomes.

The primary objective of this study was to determine the complication and re-operation rates following femoral neck fracture fixation with FNS at a major trauma centre. Secondary objectives were to assess whether fracture morphology, reduction quality, and age-related differences were associated with complications; and to compare outcomes observed in this cohort with patterns reported in the literature.

## Materials and methods

This retrospective cohort study included patients identified from electronic medical records who underwent FNS fixation for femoral neck fractures at the Department of Trauma and Orthopaedics at Sheffield Teaching Hospitals, Major Trauma Centre, United Kingdom. The study period spanned from January 2019 to December 2024. Retrospective data were collected using a consecutive sampling technique from the electronic medical records and PACS imaging platform on demographics, comorbidities, mechanism of injury, time from presentation to surgery, degree of displacement, tip-apex distance, and outcomes, with a focus on AVN and re-operation rates. The sample size comprised all eligible patients undergoing primary fixation with FNS during the study period.

Inclusion and exclusion criteria

Patients who received primary fixation with FNS for intracapsular femoral neck fractures from 2019 to 2024 at Sheffield Teaching Hospitals Major Trauma Centre were included in the study. Patients with incomplete data or any surgical fixation other than FNS were excluded from the study. Patients without a minimum radiographic and clinical follow-up of three months were excluded.

Operational definitions

Primary outcomes were the assessment of FNS fixation postoperative complications or reoperation, and secondary outcomes were to assess for the association of complications with reduction quality, age and fracture morphology, and a brief subsequent comparison of this with existing literature and alternative known fixation methods.

Procedures were performed by fellowship-trained trauma surgeons or senior trainees under direct supervision using a standardised approach and fluoroscopic confirmation of reduction. Postoperative rehabilitation was standardised with early mobilisation and weight-bearing as tolerated. All patients received radiographic follow-up for at least three months.

Data collection instruments consisted of the hospital electronic medical record system and PACS imaging platform. Radiographs were independently assessed by two orthopaedic surgeons, blinded to patient outcomes, and disagreements were resolved by consensus. Regarding fracture classification and reduction assessment, each fracture was classified according to the Garden and Pauwels systems using preoperative radiographs. Garden I-II were deemed un-displaced, and Garden III-IV were deemed displaced. Pauwels I (<30°), II (30-50°), and III (>50°) quantified vertical shear. Postoperative reduction was graded by the Garden Alignment Index (GAI), with acceptable reduction defined as valgus 160-180° on anteroposterior and 180 ± 5° on lateral views [1,2].

Data analysis

Data were analysed using standard descriptive statistics for baseline characteristics. Categorical variables such as complication rates, fracture classifications, reduction quality, and mechanism of injury were compared using the chi-square test or Fisher’s exact test where appropriate. Continuous variables such as time to theatre and tip-apex distance were assessed for normality and compared using independent-samples t-tests or Mann-Whitney U tests as required. Statistical significance was defined as p-values <0.05. Multivariate regression was planned but not performed due to an insufficient cohort.

Failure categorisation complications subsequently leading to re-operation (hardware removal, revision fixation or arthroplasty) were classified as AVN (radiographic sclerosis, subchondral collapse or deformity), mechanical failure (loss of fixation, implant cut-out or varus collapse >10°), non-union (absence of consolidation at six months), or infection (clinical diagnosis with raised inflammatory markers requiring antibiotics or washout).

## Results

A total of 70 patients underwent FNS fixation for femoral neck fractures between 2019 and 2024. Three were excluded (incomplete data), leaving 67 for analysis. The mean age was 73.6 (SD = 11.2) years (range = 31-94), and the male-to-female ratio was 30:37.

The overall complication rate was 22.4%, with a return to theatre rate of 19.4%. After excluding patients who died within 90 days postoperatively (n = 9), these rates increased to 25.9% and 22.4%, respectively. Among patients who experienced complications, 60% were attributable to AVN.

Subgroup analysis revealed a higher complication rate in patients under 65 years of age (43.8%), with a higher associated return to theatre rate of 37.5% (mainly for AVN, 5/7). In contrast, patients aged 65 years and older demonstrated lower complication and return to theatre rates (19.0% and 16.6%, respectively), predominantly for mechanical failure. Younger patients demonstrated more Pauwels III fractures (p = 0.02).

No statistically significant associations were observed between complication rates and the following variables when analysing the cohort: fracture displacement, displaced versus undisplaced (χ²(1) = 0.54 p = 0.54, Cramér’s V = 0.089); mechanism of injury, high energy versus low energy (χ²(1) = 0.19, p = 0.66, Cramér’s V = 0.054); gender, female versus male (χ²(1) = 0.23, p = 0.63, Cramér’s V = 0.059), time to theatre, or tip-apex distance (Table 1). Effect sizes for all comparisons were small, indicating a negligible association between these variables and complication risk.

**Table 1:** Association between patient injury variables and postoperative complications following Femoral Neck System fixation.

Variable	Comparison	χ²	df	P-value	Cramér’s V	Interpretation
Fracture displacement	Displaced vs. undisplaced	0.54	1	0.54	0.089	No significant association
Mechanism of injury	High-energy vs. low-energy	0.19	1	0.66	0.054	No significant association
Gender	Female vs. male	0.23	1	0.63	0.059	No significant association

Overall, 21 (31.3%) patients demonstrated Garden I-II classification, and 46 (68.7%) had Garden III-IV. On the other hand, Pauwels’ classification was identified as follows: I = 12 (17.9%), II = 34 (50.7%) and III = 21 (31.3%). An acceptable postoperative reduction (GAI criteria) was achieved in 79.1% of patients. Poor reduction correlated with high complications, but this was statistically not significant. No significant associations were found between complications and gender, mechanism, or surgical timing.

The mean time to theatre for all patients was 35.8 (SD = 51.1) hours. Patients without complications had a mean time to theatre of 39.1 (SD = 53.2) hours, compared to 33.9 (SD = 56.6) hours in those who experienced complications. Of note, patients who developed postoperative complications had a lower mean tip-apex distance of 27.36 mm (SD = 8.0 mm) compared to those without complications of 32 mm (SD 9.6 mm), although this difference did not reach statistical significance (p > 0.05).

## Discussion

Our study represents one of the larger single-centre retrospective series evaluating clinical outcomes following fixation of femoral neck fractures using the FNS. Our overall complication rate was 22.4%, increasing to 25.9% when excluding patients who died within 90 days postoperatively. AVN emerged as the most frequent complication, accounting for 60% of all adverse events. These findings provide valuable insight into the real-world performance of the FNS, particularly when contextualised against existing literature and traditional fixation methods such as cannulated cancellous screws (CCS) and dynamic hip screws (DHS) [7,8].

The FNS was developed to address some of the mechanical shortcomings of previous fixation techniques, offering enhanced angular stability and controlled dynamic compression through a minimally invasive approach [8]. Biomechanical studies have supported its superior resistance to rotational and axial loads compared to CCS, making it an attractive option, especially for unstable or vertically oriented femoral neck fractures [9]. However, despite these theoretical advantages, our complication rates, especially among younger patients, suggest that clinical performance may not yet consistently reflect biomechanical promise.

In our cohort, patients under 65 years of age experienced higher complication (43.8%) and reoperation rates (37.5%) than those aged 65 and older (19.0% and 16.6%, respectively). This finding is consistent with previous reports highlighting the unique challenges of managing femoral neck fractures in younger, more active individuals, where preservation of the native hip is prioritised, but outcomes are often suboptimal [10,11]. A systematic review by Slobogean et al. reported AVN rates of 14.3% and reoperation rates of 18.0% in young adults undergoing internal fixation for femoral neck fractures [4], while Pervez et al. reported AVN rates ranging from 10% to 30% following CCS fixation, particularly in displaced or delayed cases [7].

Our observed AVN incidence in the younger subgroup exceeds these published figures, raising concerns about the ability of FNS to mitigate this risk in this demographic [12]. While the FNS may reduce mechanical failure, it does not appear to significantly affect the biological factors that contribute to femoral head ischemia. Fracture displacement, vascular disruption at the time of injury, patient activity level, and potential delays to surgical intervention are all likely contributors [13,14].

Fracture configuration and reduction quality emerged as major influences. Vertically oriented Pauwels III and displaced Garden III-IV fractures exhibited greater AVN and mechanical failure rates. Acceptable reduction reduced, but did not eliminate, AVN. Indicating vascular compromise at injury as the main determinant.

Younger patients often had more vertical, high-energy fractures and typically bear greater physiological loads at earlier intervals than the elderly cohort, leading to AVN-dominated failures. Elderly patients presented with more oblique or osteoporotic patterns with mechanical collapse predominance. Despite this, our data exhibited complications across all ages with no statistically significant trends identified.

Comparative studies have shown mixed results regarding the efficacy of FNS. Blomfeldt et al. reported an 11.8% complication rate in a small cohort of 34 patients, with AVN being the leading cause of failure [15]. Similarly, Kim et al. found a 15.5% revision rate in a series of 71 patients, with AVN accounting for nearly half of these cases [14]. A meta-analysis by Lu-Yao et al. suggested a lower implant failure rate in the FNS group compared to CCS, but emphasised that the duration of follow-up may not have been sufficient to fully assess long-term complications such as AVN [11]. Further, Jiang et al. and Rajnish et al. found no significant difference in overall complication rates between FNS and traditional fixation devices [8,10].

The higher reoperation rate in our younger patients underscores the need to reassess the role of internal fixation in this group, despite its non-statistical significance. While preserving the femoral head is desirable, the substantial risk of failure may warrant consideration of primary arthroplasty in selected patients, particularly those with displaced fractures, poor bone quality, or limited capacity to adhere to weight-bearing restrictions [16,17]. This approach remains controversial but may offer more predictable outcomes in high-risk cases.

Our analysis showed no statistically significant association between common risk factors, such as fracture displacement, mechanism of injury, gender, or time to surgery, and complication rates. Similarly, we observed a non-significant trend of lower tip-apex distance among patients with complications, which may reflect increased surgeon precision or technical challenges in difficult cases. While tip-apex distance is a known predictor of failure in intertrochanteric fractures, its utility in the context of femoral neck fixation with the FNS remains unclear and may warrant further investigation [17-19].

Limitations of our study include its retrospective design with inherent selection bias for certain fracture patterns, modest sample size, which was non-consecutive, variable follow-up duration, lack of patient-reported outcome measures, and standardised radiographic measurements, which remain subject to variability. Surgical decision-making and patient selection were not uniform, and follow-up duration varied, potentially underestimating the true rate of late complications such as AVN. Additionally, as with any relatively new device, the learning curve associated with FNS implantation may have influenced our outcomes, particularly early in its adoption.

While this study demonstrates a notable complication and reoperation burden following FNS fixation, particularly AVN, interpretation must be cautious. Despite observed trends between complication rates, fracture morphology, and age, the study was not adequately powered to establish causality or to consider the multiple confounders. While our findings align with published concerns with respect to fixation challenges in younger patients and vertically orientated fracture patterns, definitive recommendations regarding implant selection cannot be made from this data alone and prospective studies are encouraged.

## Conclusions

Despite promising biomechanical properties, the FNS does not appear to reduce the incidence of biologically driven complications such as AVN. Our findings demonstrate a high rate of complications and reoperations in this subgroup, comparable to those seen with traditional fixation methods. Reduction quality may influence outcomes, but complications occurred across all fracture morphological types. These results highlight the importance of individualised treatment planning and raise important questions regarding the role of fixation in the management of intracapsular femoral neck fractures. Future prospective, randomised studies with longer follow-up are needed to more clearly define the risk profile and long-term efficacy of the FNS compared to established surgical fixation methods.
